# Dataset and analysis of molecular dynamics simulation of EpCAM ectodomain dimer

**DOI:** 10.1016/j.dib.2021.107403

**Published:** 2021-09-20

**Authors:** Miha Pavšič

**Affiliations:** Faculty of Chemistry and Chemical Technology, Department of Chemistry and Biochemistry, University of Ljubljana, Večna pot 113, Ljubljana SI-1000, Slovenia

**Keywords:** EpCA, Tumor marker, Molecular dynamics simulation, Structure, Residue-residue contact network

## Abstract

The data provided and described here give insight into the solution dynamics of the dimer of human EpCAM ectodomain (EpEX). As the starting point, crystal structure of EpEX non-covalent dimer was used (PDB ID 4MZV). The coordinates of solvent-embedded dimer were used to generate a topology file, which was in turn used for all-atom molecular dynamics (MD) simulation run of 20 ns length using full-system periodic electrostatics at a constant temperature of 310 K and a constant pressure of 1 atm. The MD trajectory file (part of this dataset) contains 4000 frames corresponding to recording/sampling atom positions every 5 ps. The simulation run was then analyzed in terms of root mean square deviations (RMSD) of protein atoms, and non-covalent inter-subunit interactions. The MD trajectory and analyzed data enable—in contrast to the static crystal structure—detailed analysis of solution-like protein structural dynamics and support design of EpCAM-targetting binders and structure-based analysis of EpCAM interactome.

## Specifications Table

**Table:** 

Subject	Structural Biology
Specific subject area	Computational molecular biophysics
Type of data	Structure Molecular dynamics trajectory Table Interaction network
How data were acquired	The data were acquired by molecular dynamics (MD) simulations using program NAMD 2.11 [1] running on a NVIDIA GF110 graphical processing unit (GPU). Input data were prepared using VMD 1.9.3 [2]. Data were analyzed using UCSG Chimera [3] and Cytoscape 3.8.2 [4].
Data format	Raw input: structure (pdb) and topology file (psf). Raw output: trajectory file (dcd), sampled structure snapshots (pdb). Analyzed: rmsd values (xlsx), residue-residue contact networks (pdf), network on non-covalent interactions (cys).
Parameters for data collection	Protein model (EpCAM ectodomain dimer) was embedded in a water cube with periodic boundary conditions, system was electro-neutral. For simulation CHARMM22 force field was used, and simulation was run at 1 atm and 310 K.
Description of data collection	Molecular dynamics simulation of the EpCAM ectodomain dimer was performed using NAMD 2.11 [1]. The resulting trajectory file was used to prepare structure snapshots in pdb format, as well as to calculate frequency of inter-subunit interactions involving specific residues during the timecourse of the simulation and RMSD values of C_α_ atoms of the simulated prorein model.
Data source location	Institution: University of Ljubljana, Faculty of Chemistry and Chemical Technology City: Ljubljana Country: Slovenia
Data accessibility	Repository name: Mendeley Data Data identification number: 10.17632/44p89zc67y.1 Direct URL to data: http://dx.doi.org/10.17632/44p89zc67y.1
Related research article	T. Žagar, M. Pavšič, A. Gaber, Destabilization of EpCAM dimer is associated with increased susceptibility towards cleavage by TACE, PeerJ. 9 (2021) e11484. 10.7717/peerj.11484.

## Value of the Data

• The data are useful for detailed structural analysis of tumor marker EpCAM ectodomain dimer. In contrast to the static crystal structure, the data mimick structural dynamics of protein in the solution.
• All-atom protein molecular dynamics simulations in nanosecond scale are inherently time-consuming to calculate. This dataset enables structural biologists to use a pre-calculated molecular dynamics trajectory of EpCAM ectodomain dimer.
• Data provide insight into which regions of the EpCAM ectodomain dimer are more structurally flexible than the others, and which inter-subunit interactions are pivotal for dimer stability.
• Data can be used to extract intra-subunit residue–residue interactions at atomic resolution providing information on EpCAM molecular biophysics and protein biophysics in general.
• Ensemble of structure snaphots can be used as a model for phasing by molecular replacement during crystal structure solution of EpCAM ectodomain from other species or EpCAM-related molecules, and as models in structural studies by other methods.
• This dataset can be used in the design of molecules specifically targetting EpCAM (potential therapeutics), or to devise mutations aimed at interfering with EpCAM function, stability and/or oligomeric state (research purpose).

## 1 Data Description

The data described here are derived from molecular dynamics (MD) simulation of a native-like dimer of ectodomain of epithelial cell adhesion molecule (EpCAM). The MD trajectory file, which is part of this dataset, corresponds to a 20 ns all-atom simulation and is an extension of the MD simulation described in Ref. [5]. Supplied are initial coordinates and topology of the simulated system, output energies (frequency of 0.2 ps) and trajectory with atom coordinates (frequency of 5 ps), and structure snaphots in pdb format of the dimer and subunits (frequency of 200 ps). The dataset also includes a file listing root mean square deviation (rmsd) values for each residue, and a non-covalent inter-subunit interactions network (Cytoscape format). The Cytoscape file containing residue-residue interaction network contains several rows describing the nodes (residues), including shared name (three-letter residue code with residue number and chain ID), SS (secondary structure), and kdHydrophobicity (hydrophobicity assigned according to Kyte-Doolittle scale). The weight in the edge table (table of residue-residue interactions) corresponds to the frequency of the observed contact during the simulation (in the range from 0 to 1, with 1 corresponding to contact in 100% of simulation frames).

**Fig. 1:**
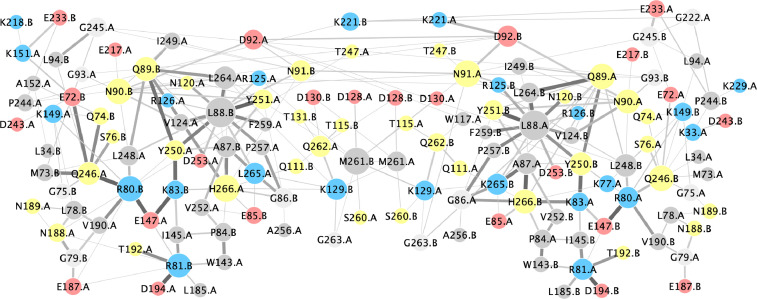
Residue interaction network. Shown are non-covalent interactions between the two subunits of the EpEX ectodomain dimer during MD simulation. Edge thickness and color coresponds to observed frequency of the interaction during trajectory—thicker and darker line corresponds to higher frequency. Node color corresponds to charge: positively charged residues as blue (Lys, Arg), negatively charged residues as red (Glu, Asp), polar residues as light green, and hydrophobic as grey. Node size corresponds to degree (number of different interactions). Modified from [5] by including data from extended simulation time and manually rearranging the residue nodes for better readability.

**Fig. 2:**
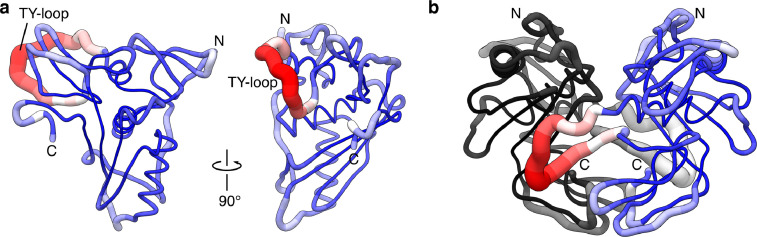
RMSD of C_α_ atoms mapped to initial structure of EpCAM (**a**) subunit, and (**b**) ecotodomain dimer (PDB ID 4MZV). Broader tube and red color corresponds to large RMSD values while narrower tube and blue color corresponds to lower RMSD values. In the dimer one subunit is colored as in gray–black gradient which corresponds to red–blue of the other subunit. Labeled are N- and C-termini, plus the first loop of the thyroglobulin type 1 (TY) of EpCAM.

All mentioned files are listed in Table 1. The inter-subunit interactions are depicted as residue-residue interaction network in the Fig. 1, and RMSD values mapped to the initial structure are shown in Fig. 2.

**Table 1:** Data files provided for the MD simulation.

Sub-folder name	File name	Description
input	EpEX_x4mzv.pdb	input protein dimer structure
	EpEX_x4mzv_wbi.pdb	coordinates of simulated system including water molecules and sodium ions
	EpEX_x4mzv_wbi.psf	topology file for the simulated system
output	EpEX_x4mzv_eq.xst	energies
	EpEX_x4mzv_eq-wrapped.dcd	trajectory file (centered on protein)
output/ pdb_snapshots_dimer	EpEX_x4mzv-frame_$i.pdb	snapshots of dimer coordinates ($i = frame number, starting at 0)
output/ pdb_snapshots_subunit	EpEX_x4mzv-segA-frame_$i.pdb EpEX_x4mzv-segB-frame_$i.pdb	snapshots of subunit coordinates ($i = frame number, starting at 0; segA, segB = segment ID)
analysis	EpEX_x4mzv-subunit_rmsd.xlsx	RMSD values (per residue)
	EpEX_x4mzv-subunit_interactions.cys	Cytoscape file containing non-covalent inter-subunit interactions
	EpEX_x4mzv-subunit_interactions.pdf	PDF output of Cytoscape file containing non-covalent inter-subunit interactions

## 2 Experimental Design, Materials and Methods

Directories and file described below are part of the master file **EpEX_4mzv-MD_dataset.zip** deposited at Mendeley Data.

### 2.1 Preparation of input topology files

As the starting structure EpEX crystal structure was used (PDB ID 4MZV) [6]. The structure contains one polypeptide chain in the asymmetric unit, and the EpEX dimer was constructed by applying a symmetry operation (rotation around C2 axis) using UCSF Chimera [3]. Chains were labeled A and B, respectively, and from both of them the N-terminal pyroglutamate residue (pyroGlu24) was removed. This initial dimer structure (file: input/EpEX_x4mzv.pdb) was used to generate the all-atom pdb (file: input/EpEX_x4mzv_wbi.pdb) and topology (file: input/EpEX_x4mzv_wbi.psf) using VMD 1.8.3 (http://www.ks.uiuc.edu/Research/vmd/) and the psfgen plugin [2]. During this procedure, histidine residues were listed as HSE (neutral His, proton on NE2), 20 Å water margin was added on each side of the dimer (giving a box of approximately 100 × 100 × 100 Å), and the system was neutralized by adding sodium ions. The all-atom pdb file contains 7608 protein atoms (segments SEGA and SEGB, corresponding to the two subunits), 89,688 water atoms (29,896 water molecules) and 4 sodium ions giving together a total of 97,300 atoms/ions.

### 2.2 Molecular dynamics simulation

MD simulation runs were performed using NAMD 2.11 [1] (http://www.ks.uiuc.edu/Research/namd/) running on a NVIDIA GF110 graphical processing unit (GPU) on a 64-bit Linux system. Following initial minimization (1000 steps of 2 fs), the water molecules and ions were allowed to move freely for 5000 steps (each 2 fs) while the protein atoms were kept at fixed positions. This step allowed water molecules to enter small cavities and to rearrange themselves in a real solution-like manner, thereby preventing introduction of artefacts during the production run. After this step the system was remeasured, and the new dimensions used to define the size of the production system. The production run of 20 ns length was performed under periodic boundary conditions where full-system periodic electrostatics were used, again using a timestep of 2 fs for recalculation of energy and forces. Simulations of similar length were already shown to be relevant to explore local structure fluctuations or conformational changes in other dimers with similar a dimer-to-monomer dissociation constant in (sub)nanomolar range, for example of the human prion protein dimer [7] and tubulin dimer [8]. For both initial minimization and final production run CHARMM22 forcefield parameters [9], [10] were used. Temperature was kept constant at 310 K using Langevin dynamics, and pressure at 1 atm using Langevin piston. The atom positions were recorded with a frequency of 5 ps giving a final trajectory file of 4000 frames, and the energy was recorded with a frequency of 0.2 ps (file: output/EpEX_x4mzv_eq.xst). The trajectory file was wrapped using PBCTools 2.8 (part of VMD) to center on the protein part of the system (file: output/EpEX_x4mzv_eq-wrapped.dcd).

### 2.3 Generation of structure snaphots from MD trajectory

The wrapped trajectory and corresponding topology file were loaded in VMD 1.8.3 and used to generate structure snapshots of the dimer and separate subunits; for each, 100 snapshots were generated corresponding to every 40th frame of the trajectory. The files are collected in separate folders: output/pdb_snapshots_dimer/EpEX_x4mzv-frame_$i.pdb for the dimer ($i correspondis to frame number starting from 0), and output/pdb_snapshots_dimer/EpEX_x4mzv-segX-frame_$i.pdb for the subunits (X corresponds to A or B, and $i correspondis to frame number starting from 0).

### 2.4 Calculation of RMSD values

Root mean square deviation of backbone atoms C_α_ was calculated by using pdb structure snapshots of the two subunits, and superimposing them using Theseus 3.3.0 [11,12]. The per residue RMSD values are listed in the file analysis/EpEX_x4mzv-subunit_rmsd.xlsx.

### 2.5 Inter-subunit contacts analysis

Non-covalent contacts between the subunits of the EpEX dimer were analyzed using UCSF Chimera [3] connected to Cytoscape 3.8.2 [4] with the StructureViz2 plugin [13]. Each 10th frame of the MD trajectory was analyzed, and to each observed residue-residue interaction a fraction of frames in which it was present was assigned. For contact detection default parameters were used (VdW overlap ≥ –0.4 Å).

## CRediT authorship contribution statement

**Miha Pavšič:** Conceptualization, Methodology, Formal analysis, Investigation, Data curation, Writing – original draft, Writing – review & editing, Visualization.

## Declaration of Competing Interest

The author declares that he has no competing financial interests or personal relationships which have or could be perceived to have influenced the work reported in this article.

## Appendix Supplementary materials

Image, application 1

## References

[1] Phillips J.C., Hardy D.J., Maia J.D.C., Stone J.E., Ribeiro J.V., Bernardi R.C., Buch R., Fiorin G., Hénin J., Jiang W., McGreevy R., Melo M.C.R., Radak B.K., Skeel R.D., Singharoy A., Wang Y., Roux B., Aksimentiev A., Luthey-Schulten Z., Kalé L.V., Schulten K., Chipot C., Tajkhorshid E. (2020). Scalable molecular dynamics on CPU and GPU architectures with NAMD. J. Chem. Phys..

[2] Humphrey W., Dalke A., Schulten K. (1996). VMD: visual molecular dynamics. J. Mol. Gr..

[3] Pettersen E.F., Goddard T.D., Huang C.C., Couch G.S., Greenblatt D.M., Meng E.C., Ferrin T.E. (2004). UCSF Chimera–a visualization system for exploratory research and analysis. J. Comput. Chem..

[4] Shannon P., Markiel A., Ozier O., Baliga N.S., Wang J.T., Ramage D., Amin N., Schwikowski B., Ideker T. (2003). Cytoscape: a software environment for integrated models of biomolecular interaction networks. Genome Res..

[5] Žagar T., Pavšič M., Gaber A. (2021). Destabilization of EpCAM dimer is associated with increased susceptibility towards cleavage by TACE. PeerJ.

[6] Pavšič M., Gunčar G., Djinović-Carugo K., Lenarčič B. (2014). Crystal structure and its bearing towards an understanding of key biological functions of EpCAM. Nat. Commun..

[7] Sekijima M., Motono C., Yamasaki S., Kaneko K., Akiyama Y. (2003). Molecular dynamics simulation of dimeric and monomeric forms of human prion protein: insight into dynamics and properties. Biophys. J..

[8] Eren E., Watts N.R., Sackett D.L., Wingfield P.T. (2021). Conformational changes in tubulin upon binding Cryptophycin-52 reveal its mechanism of action. J. Biol. Chem..

[9] MacKerell A.D., Bashford D., Bellott M., Dunbrack R.L., Evanseck J.D., Field M.J., Fischer S., Gao J., Guo H., Ha S., Joseph-McCarthy D., Kuchnir L., Kuczera K., Lau F.T., Mattos C., Michnick S., Ngo T., Nguyen D.T., Prodhom B., Reiher W.E., Roux B., Schlenkrich M., Smith J.C., Stote R., Straub J., Watanabe M., Wiórkiewicz-Kuczera J., Yin D., Karplus M. (1998). All-atom empirical potential for molecular modeling and dynamics studies of proteins. J. Phys. Chem. B.

[10] Mackerell A.D., Feig M., Brooks C.L. (2004). Extending the treatment of backbone energetics in protein force fields: limitations of gas-phase quantum mechanics in reproducing protein conformational distributions in molecular dynamics simulations. J. Comput. Chem..

[11] Theobald D.L., Wuttke D.S. (2008). Accurate structural correlations from maximum likelihood superpositions. PLoS Comput. Biol..

[12] Theobald D.L., Steindel P.A. (2012). Optimal simultaneous superpositioning of multiple structures with missing data. Bioinformatics.

[13] Morris J.H., Huang C.C., Babbitt P.C., Ferrin T.E. (2007). structureViz: linking Cytoscape and UCSF Chimera. Bioinformatics.

